# Electrospun Fibers of Biocompatible and Biodegradable Polyesters, Poly(Ethylene Oxide) and Beeswax with Anti-Bacterial and Anti-Fungal Activities

**DOI:** 10.3390/ma16134882

**Published:** 2023-07-07

**Authors:** Selin Kyuchyuk, Dilyana Paneva, Nevena Manolova, Iliya Rashkov, Daniela Karashanova, Mladen Naydenov, Nadya Markova

**Affiliations:** 1 Laboratory of Bioactive Polymers, Institute of Polymers, Bulgarian Academy of Sciences, Acad. G. Bonchev St., Bl. 103A, BG-1113 Sofia, Bulgaria; selin.erdinch@polymer.bas.bg (S.K.); manolova@polymer.bas.bg (N.M.); 2 Institute of Optical Materials and Technologies, Bulgarian Academy of Sciences, Acad. G. Bonchev St., Bl. 109, BG-1113 Sofia, Bulgaria; dkarashanova@yahoo.com; 3 Department of Microbiology, Agricultural University, BG-4000 Plovdiv, Bulgaria; mladen@au-plovdiv.bg; 4 Institute of Microbiology, Bulgarian Academy of Sciences, Acad. G. Bonchev St., Bl. 26, BG-1113 Sofia, Bulgaria; markn@bas.bg

**Keywords:** core–sheath fibers, core–shell fibers, electrospinning, 8-hydroxyquinoline derivatives, anti-bacterial activity, anti-fungal activity, phytopathogenic microorganisms, phytopathogenic fungi, beneficial microorganisms

## Abstract

Fibrous materials composed of core–sheath fibers from poly(ethylene oxide) (PEO), beeswax (BW) and 5-nitro-8-hydroxyquinoline (NQ) were prepared via the self-organization of PEO and BW during the single-spinneret electrospinning of a homogeneous blend solution of the partners. Additionally, the application of the same approach enabled the preparation of fibrous materials composed of core–double sheath fibers from PEO, poly(L-lactide) (PLA) and NQ or 5-chloro-7-iodo-8-hydroxyquinoline (CQ), as well as from PEO, poly(ε-caprolactone) (PCL) and NQ. The consecutive selective extraction of BW and of the polyester with hexane and tetrahydrofuran, respectively, evidenced that core–double sheath fibers from PEO/polyester/BW/drug consisted of a PEO core, a polyester inner sheath and a BW outer sheath. In order to evaluate the possibility of the application of fibrous materials from PEO/BW/NQ, PEO/PLA/BW/NQ, PEO/PCL/BW/NQ and PEO/PLA/BW/CQ for plant protection, microbiological studies were performed using both phytopathogenic microorganisms (*Pseudomonas corrugata*, *Fusarium graminearum* and *Fusarium avenaceum*) and beneficial microorganisms (*Pseudomonas chlororaphis*, *Bacillus amyloliquefaciens* and *Trichoderma asperellum*). It was found that the fibrous materials had anti-bacterial and anti-fungal activity against both phytopathogenic and beneficial microorganisms. This is the first report on the activity of fibrous materials loaded with 8-hydroxyquinoline derivatives not only against phytopathogenic but also against beneficial microorganisms that are of importance in agriculture.

## 1. Introduction

The fibrous materials prepared via electrospinning are considered extremely promising, with most of the studies on their applicability being in fields such as pharmacy, medicine, and ecology [1]. However, studies on the applicability of electrospun materials in agriculture, more precisely for plant protection, are scarce [2,3]. These materials can serve as carriers of agropharmaceuticals (e.g., anti-bacterial and/or anti-fungal agents, fertilizer, and hormones) [4,5]. From a technological viewpoint, electrospinning is very attractive since it enables one-step application of an agropharmaceutical or beneficial microorganism embedded in a polymer carrier on the green part of the plants and on pruning plant wounds, as well as for seed coating [3,4,6].

It is known that 8-hydroxyquinoline and its derivatives exhibit potent anti-bacterial and anti-fungal activity [7,8,9], and they are also effective against phytopathogenic microorganisms [10]. Therefore, in the present study, we focused on two 8-hydroxyquinoline derivatives: 5-nitro-8-hydroxyquinoline (NQ) and 5-chloro-7-iodo-8-hydroxyquinoline (CQ).

Considering the potential application of fibrous materials as carriers of NQ or CQ for plant protection, we chose biocompatible aliphatic polyesters poly(L-lactide) (PLA) and poly(ε-caprolactone) (PCL), poly(ethylene oxide) (PEO) and the natural product beeswax (BW). In addition to being biocompatible, PLA and PCL are also hydrolytically degradable and biodegradable, and are considered to be the polymers that will replace conventional synthetic polymers [11]. PEO is an amphiphilic polymer that is widely used in medicine and pharmacy [12]. BW is a hydrophobic natural product composed of saturated hydrocarbons, fatty acids, fatty alcohols and esters of fatty acids and fatty alcohols [13,14].

In a previous study, we demonstrated the possibility of preparing materials consisting of PEO/BW core–sheath fibers (PEO core and BW sheath) loaded with NQ via the single-spinneret electrospinning of a homogeneous stable blend solution of PEO/BW/NQ in the common solvent chloroform [15]. PEO/BW/NQ fibrous materials have anti-bacterial and anti-fungal activity against human pathogenic microorganisms as evidenced by the microbiological studies against the bacteria *Staphylococcus aureus* (*S. aureus*) and *Escherichia coli* (*E. coli*), and the fungus *Candida albicans* (*C. albicans*). In the following study, in order to improve the mechanical behavior of the fibrous materials based on PEO, BW and NQ, PLA was added to the spinning solution, and core–double sheath fibers (PEO core, PLA inner sheath and BW outer sheath) were obtained [16]. It has been found that PEO/PLA/BW/NQ mats have anti-bacterial activity against the human pathogenic bacteria *S. aureus*, *E. coli* and *Pseudomonas aeruginosa*, and anti-fungal activity against the fungus *C. albicans*. The formation of core–sheath or core–double sheath fibers has been attributed to phase separation between the components during the electrospinning process. The reasons for the phase separation have been found to lie in the incompatibility between the partners, the difference between their molar masses, and the air hydrophobicity in the vicinity of the spinning jet surface. The core–sheath and core–double sheath architecture has been evidenced via transmission electron microscopy as well as via the selective extraction of the sheath(s) or core components [15,16]. There are data on the use of core–sheath fibers as drug carriers that provide the preservation of the bioactivity of embedded environmentally sensitive bioactive substances, as well as their sustained and controlled release [17]. However, such data are lacking for core–double sheath fibers, with the exception of our data on the anti-microbial activity of PEO/PLA/BW/NQ fibers against human pathogenic microorganisms [16]. It is to be expected that the presence of a second sheath from the aliphatic polyester PLA might ensure a sustained drug release as compared to the core–sheath fibers from PEO/BW. Furthermore, it might be assumed that the polyester nature and molar mass might affect the amount of the released drug over time as well. That is the reason for the preparation, in the present study, of a set of core–sheath (PEO/BW) and core–double sheath fibers (PEO/PLA/BW or PEO/PCL/BW) containing NQ or CQ, in addition to the evaluation of the effect of the fibers’ architecture in terms of NQ release, and of the ability to display the anti-microbial activity of the incorporated NQ or CQ. We have selected phytopathogenic microorganisms and beneficial microorganisms in order to assess the potential of the prepared core–sheath and core–double sheath fibers to be applied for plant protection. To the best of our knowledge, such information has not been reported to date.

In the present study, we focused on *Pseudomonas corrugata* (*P. corrugata*)*, Fusarium graminearum* (*F. graminearum*) and *Fusarium avenaceum* (*F. avenaceum*) as representatives of phytopathogenic microorganisms, and on *Pseudomonas chlororaphis* (*P. chlororaphis*), *Bacillus amyloliquefaciens* (*B. amyloliquefaciens*) and *Trichoderma asperellum* (*T. asperellum*) as representatives of beneficial microorganisms. We selected these pathogenic microorganisms since they were considered to be one of the most widespread phytopathogens, leading to a significant decrease in the crop yield and quality. In addition, some of these phytopathogens produce mycotoxins. *P. corrugata* is a Gram-negative bacterium that causes necrosis and/or hollowing of the pith of the stem in tomatoes [18]. *F. graminearum* is a filamentous fungus that causes head blight (scab) diseases in cereal crops and ear rot in maize, and leads to the contamination of the grain with various mycotoxins [19,20]. *F. avenaceum* is a filamentous fungus that causes diseases such as Fusarium seedling blight and Fusarium head blight on barley, and this fungus is characterized by the production of mycotoxins [21]. Regarding the selected beneficial microorganisms, they were selected since these species are one of the most commonly used beneficial microorganisms included in a number of commercial products. *P. chlororaphis* is a beneficial Gram-negative bacterium, and it is applied as a beneficial microorganism in agriculture since it produces metabolites that are effective against a wide range of phytopathogens, insects and nematodes [22]. *B. amyloliquefaciens* is a beneficial Gram-positive bacterium and is used in agriculture against root phytopathogens (e.g., *Alternaria* and *Fusarium*) [23]. In addition, its metabolites serve as plant growth promoters, probiotics and bioremediation agents. *T. asperellum* is a beneficial rhizosphere filamentous fungus and is used in agriculture against a wide range of phytopathogens. *T. asperellum* is considered to be the best candidate as a beneficial microorganism for green agriculture, since, in addition to fighting phytopathogens, it also has a wide biofertilization and biostimulatory potential [24].

The aim of the present study was to evaluate, in vitro, the behavior of PEO/BW/NQ, PEO/PLA/BW/NQ, PEO/PCL/BW/NQ and PEO/PLA/BW/CQ fibrous materials in contact with some phytopathogens and beneficial microorganisms. Not yet reported PEO/PCL/BW/NQ and PEO/PLA/BW/CQ mats were prepared via the single-spinneret electrospinning of homogeneous blend solutions of the partners as typical representatives of a PEO/polyester/BW system with desired characteristics. Consecutive selective extraction with hexane and tetrahydrofuran was applied in order to evidence the composition of the core, and of the inner and outer sheath of the core–double sheath fibers. The behavior of PEO/BW/NQ, PEO/PLA/BW/NQ, PEO/PCL/BW/NQ and PEO/PLA/BW/CQ mats in contact with phytopathogens (the bacterium *P. corrugata*, and the fungi *F. graminearum* and *F. avenaceum*) and beneficial microorganisms (the bacteria *P. chlororaphis* and *B. amyloliquefaciens* and the fungus *T. asperellum*) was microbiologically tested.

## 2. Materials and Methods

### 2.1. Materials

Poly(ethylene oxide) (PEO, Badimol-M, Dimitrovgrad, Bulgaria) was used, with a viscosity average molar mass of 600,000 g/mol as determined in distilled water at 30 °C using a Ubbelohde viscometer with the equation: $[\eta ]=1.25{\times 10}^{-4}{\times M\eta }^{0.78}$ [25]. Poly(L-lactide) was used (PLA, Ingeo™ Biopolymer 4032D, NatureWorks LLC—Minneapolis, MN, USA; M_w_ = 259,000 g/mol; M_w_/M_n_ = 1.94; as determined via size-exclusion chromatography using polystyrene standards). Poly(ε-caprolactone) (PCL, CAPA 6800) was supplied by Solvay Interox, Warrington, UK (M_n_ = 69,000 g/mol; M_w_/M_n_ = 1.74). Beeswax with a purity corresponding to the European Pharmacopoeia was bought from Chemax Pharma Ltd., Sofia, Bulgaria; and chloroform, hexane, tetrahydrofuran (THF), dimethyl sulfoxide (DMSO)—from Merck (Darmstadt, Germany). 5-Nitro-8-hydroxyquinoline (NQ) and 5-chloro-7-iodo-8-hydroxyquinoline (CQ) were purchased from Sigma-Aldrich, Buchs, Switzerland. Na_2_HPO_4_, and KH_2_PO_4_ were purchased from Merck (Darmstadt, Germany). All of the abovementioned chemicals are reagents for analytical applications and were used as received. The phytopathogenic microorganisms *Pseudomonas corrugata* PPB21, *Fusarium graminearum* PPF 9 and *Fusarium avenaceum* PPF107, and beneficial microorganisms *Bacillus amyloliquefaciens* B3, *Pseudomonas chlororaphis* P1 and *Trichoderma asperellum* T6 were used. All of the microorganisms were obtained from the collection of Biodinamika Ltd., Plovdiv, Bulgaria. The microbial growth medium for the bacteria was tryptic soy agar (Biolife, Milan, Italy). The microbial growth medium for the fungi was potato dextrose agar (Merck, Darmstadt, Germany).

### 2.2. Preparation and Characterization of Electrospun Fibrous Materials from PEO/BW/NQ, PEO/PLA/BW/NQ, PEO/PCL/BW/NQ u PEO/PLA/BW/CQ

PEO/BW/NQ fibrous materials were prepared via the single-spinneret electrospinning of a homogeneous blend solution using the methodology described in [18]. The PEO/BW/NQ mats used in the present study were prepared at PEO/BW = 70/30 or 60/40 (*w/w*) and NQ content of 10 wt.% in respect to the total weight of the solids. PEO/PLA/BW/NQ mats were prepared via the single-spinneret electrospinning of a homogeneous blend solution as described in [19]. The PEO/PLA/BW/NQ mats used in the present study were prepared at PEO/PLA/BW = 70/15/15 or 60/20/20 (*w/w/w*) and NQ content of 10 wt.% in respect to the total weight of the solids. Using the same conditions, PEO/PCL/BW/NQ and PEO/PLA/BW/CQ were prepared as well at PEO/polyester/BW = 60/20/20 (*w/w/w*) and drug (NQ or CQ) content of 10 wt.% in respect to the total weight of the solids.

The morphology of the fibers in the electrospun mats and the core–sheath or core–double sheath architecture of the fibers were evaluated via scanning electron microscopy (SEM; Philips 515 SEM, Tokyo, Japan) and transmission electron microscopy (TEM; JEM 2100 TEM, JEOL Co., Ltd., Tokyo, Japan, operating voltage 200 kV), respectively. At least 30 fibers from the SEM images were used for the estimation of the mean fiber diameters using Image J 1.52q software. Concerning the TEM analyses, individual fibers were deposited on a copper grid and then observed via TEM.

In order to assess the composition of the core, inner and outer sheath of core–double sheath fibers, a systematic approach was applied. It consisted of the consecutive selective extraction of BW with hexane and of the polyester with THF. For that purpose, 40 mg of PEO/polyester/BW/NQ mats was immersed in 30 mL hexane at r.t. for 24 h. After that, the fibrous specimens were removed from the hexane and dried at r.t. for 24 h. The weight loss of the samples was determined gravimetrically. A part of the samples was subjected to analysis via SEM and TEM, as well as for the determination of the water contact angle. The other part of the samples was immersed in 30 mL THF at r.t. for 4 h. After that, the fibrous specimens were removed from THF and dried at r.t. for 24 h. Then, they were analyzed in terms of weight loss and water contact angle via TEM and SEM.

The static water contact angle of the fibrous materials was determined by measuring the contact angle of 10 different water drops deposited on each sample using an Easy Drop DSA20E Krüss GmbH apparatus (Hamburg, Germany; volume of the water drop—6.25 µL). The water contact angle values were estimated using the Easy Drop DSA20E.

The release of NQ from PEO60/BW40/NQ, PEO60/PCL20/BW20/NQ and PEO60/PLA20/BW20/NQ was evaluated in vitro using a phosphate buffer (Na_2_HPO_4_/KH_2_PO_4_) solution with pH 7.4 (ionic strength 0.1) at 37 °C. The mats (3 mg; 10 × 10 mm) were immersed in 100 mL of the buffer solution and the samples were thermostated in a shaker bath (Julabo, Seelbach, Germany) at 37 °C using a cell [15] for the fixation of the fibrous materials. The release of NQ was evaluated by withdrawing aliquots from the solution at certain time intervals and recording their absorbance using a DU 800 UV–vis spectrophotometer (Beckman Coulter, Brea, CA, USA) at a wavelength of 447 nm. The amount of released NQ over time was calculated using calibration curves in phosphate buffer (Na_2_HPO_4_/KH_2_PO_4_, pH 7.4, ionic strength 0.1) with a correlation coefficient of R~0.999. The data were average values from three measurements.

### 2.3. In Vitro Assessment of the Behavior of Electrospun Materials in Contact with Phytopathogenic and Beneficial Microorganisms

The behavior of fibrous materials from PEO/BW/NQ, PEO/PLA/BW/NQ, PEO/PCL/BW/NQ and PEO/PLA/BW/CQ, as well as that of non-drug-containing control electrospun mats or filter paper disks pre-dropped with a solution of NQ or CQ in DMSO in contact with the studied microorganisms, was assessed in vitro using the disk diffusion method [26] with some modifications. The disk specimens had a diameter of 10 mm. These specimens were placed in Petri dishes (diameter of 90 mm) preliminarily inoculated with a 0.1 mL suspension of bacterial or fungal culture (1 × 10^5^ cells/mL for *P. corrugata*, *P. chlororaphis* and *B. amyloliquefaciens*; or 1 × 10^4^ cells/mL for *F. graminearum*, *F. avenaceum and T. asperellum*). The Petri dishes with the specimens were incubated at 28 °C for 72 h in the case of the bacteria and 144 h in the case of the fungi, and after that, the inhibition zones around each disk were estimated using Image J 1.52q software.

## 3. Results and Discussion

### 3.1. Composition, Morphology and Structural Peculiarities of PEO/BW/NQ, PEO/PLA/BW/NQ, PEO/PCL/BW/NQ and PEO/PLA/BW/CQ Fibers

**Figure 1:**
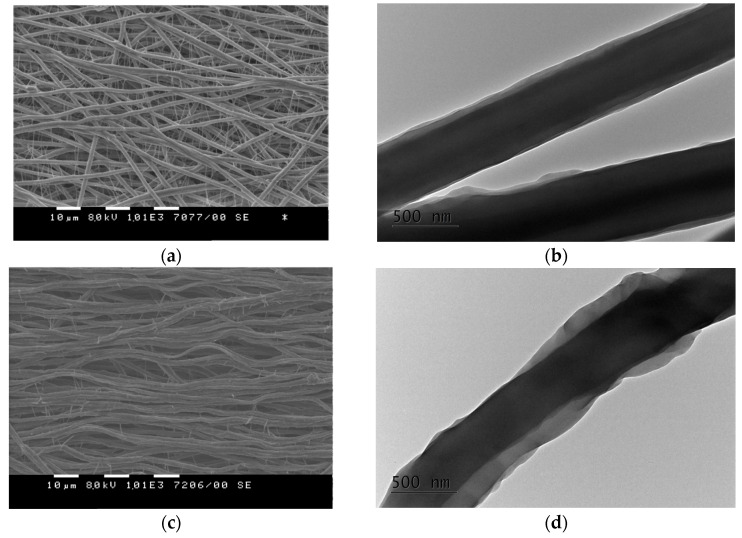
SEM (**a,c**; magnification: ×1000, scale bar: 10 µm) and TEM (**b,d**; magnification: ×10,000, scale bar: 500 nm) micrographs of fibers from PEO60/PLA20/BW20/CQ (**a**,**b**) and PEO60/PCL20/BW20/NQ (**c**,**d**).

The materials composed of core–sheath or core–double sheath fibers loaded with the bioactive compound (NQ or CQ of 10 wt.% in respect to the total weight of the solids) were prepared via the single-spinneret electrospinning of the homogeneous blend solutions. The fibrous materials of core–sheath fibers from PEO/BW = 70/30 or 60/40 (*w/w*) loaded with NQ were prepared as described in [15]. Further, these mats are denoted as PEO70/BW30/NQ and PEO60/BW40/NQ, respectively. Materials consisting of core–double sheath fibers from PEO, PLA, BW and NQ PEO/PLA/BW = 70/15/15 or 60/20/20, (*w/w*/*w*) denoted as PEO70/PLA15/BW15/NQ and PEO60/PLA20/BW20/NQ, respectively, were prepared as described earlier [16]. Moreover, not yet reported fibrous materials composed of core–double sheath fibers from PEO, PCL, BW and NQ: PEO/PCL/BW = 60/20/20 (*w/w/w*), denoted as PEO60/PCL20/BW20/NQ; as well as those from PEO, PLA, BW and CQ [PEO/PLA/BW = 60/20/20 (*w/w/w*)], denoted as PEO60/PLA20/BW20/CQ, were also prepared via the single-spinneret electrospinning of homogeneous blend solutions. The obtained set of fibrous materials composed of core–sheath or core–double sheath fibers were subjected to microbiological screening for evaluation of their behavior in contact with phytopathogenic or beneficial microorganisms. SEM analyses showed that the incorporation of NQ or CQ in PEO/BW or PEO/polyester/BW resulted in obtaining fibers with whiskers along the main fibers (Figure 1a,c). The mean diameter of the main fibers from PEO60/PLA20/BW20/CQ mat was 1.90 ± 0.19 µm, and that of the whiskers was 0.40 ± 0.07 µm. For the PEO60/PCL20/BW20/NQ mat, the mean diameter of the main fibers was 1.80 ± 0.20 µm, and that of the whiskers was 0.60 ± 0.07 µm. The formation of the whiskers was attributed to the ionic imbalance on the charged surface during the electrospinning jet induced by the presence of NQ or CQ in the spinning solution. This led to the ejection of thinner jets from the main jet and to whiskers’ formation. The obtained results are in accordance with data for the electrospinning of solutions that contain non-ionogenic polymer and low-molecular-weight salt [27,28]. TEM analyses revealed that PEO60/PLA20/BW20/CQ and PEO60/PCL20/BW20/NQ fibers had core–double sheath structure (Figure 1b,d).

Previously, by applying extraction with hexane or water, it was evidenced that the core–sheath PEO/BW fibers consisted of a PEO core and BW sheath [15], and the core–double sheath PEO/PLA/BW fibers of a PEO core, PLA inner sheath and BW outer sheath [16]. In order to elucidate the structure of the fibers from the PEO/PLA/BW/NQ, PEO/PCL/BW/NQ and PEO/PLA/BW/CQ systems in the present study, we applied a consecutive selective extraction of BW and polyester (PLA or PCL) from PEO/polyester/BW loaded with NQ or CQ with hexane or THF. Hexane is a good solvent of BW and a poor solvent of polyester, PEO, NQ, and CQ, as evidenced by the experiments on the solubility of the individual components in hexane. Thus, the immersion of the fibrous materials consisting of core–double sheath fibers in hexane would lead to the elimination of BW from the fibers. In turn, THF is a good solvent for PLA and PCL and a poor solvent for PEO, BW, and NQ or CQ. Hence, the use of this solvent would lead to the dissolution of PLA or PCL from the fibers. The consecutive extraction of BW and PLA from PEO60/PLA20/BW20/NQ with hexane and THF did not alter the yellow color of the fibrous material.

**Figure 2:**
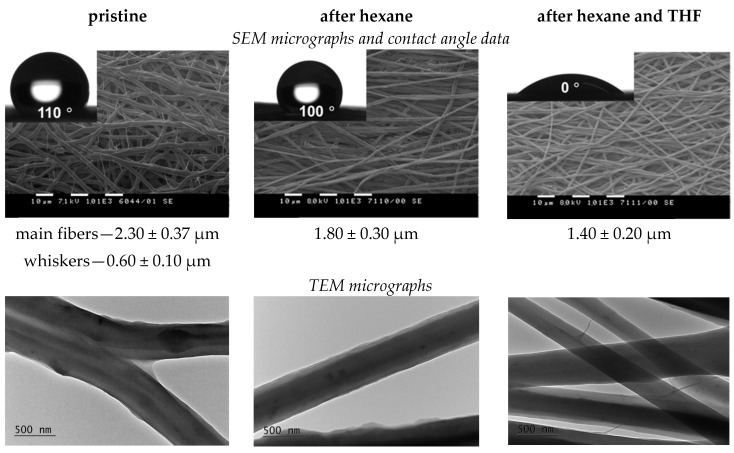
SEM and TEM micrographs of a mat and of a single fiber, respectively, from PEO60/PLA20/BW20/NQ fibrous materials before and after consecutive extraction with hexane and THF. The mean fiber diameter values are given as estimated via SEM; inset—water contact angle values. Magnification of the SEM micrographs: ×1000, scale bar: 10 µm; and of the TEM micrographs: ×10,000, scale bar: 500 nm.

This indicates that a great part of NQ remained incorporated in the fibrous material after the extractions. A similar behavior was observed in the case of PEO/PCL/BW/NQ and PEO/PLA/BW/CQ mats. PEO/PLA/BW/NQ, PEO/PCL/BW/NQ and PEO/PLA/BW/CQ fibrous materials were hydrophobic, as evidenced by the data from the determination of the water contact angle, and the water contact angle value being ca. 110°. This indicates that there was a hydrophobic component—BW and/or polyester—on the fiber’s surface. The extraction with hexane did not lead to a significant alteration of the water contact angle value, as seen from Figure 2. The decrease in the mean diameter of the fibers and the disappearance of whiskers (Figure 2), as well as the determined weight loss of the fibrous material, evidenced that BW had been eliminated from the fibers after the extraction of the mat with hexane. The theoretical weight loss calculated, assuming that the entire amount of BW had been dissolved in hexane, was 18%. The experimentally determined value of 20% slightly exceeded the theoretical one, thus indicating that, in addition to BW dissolution, the extraction with hexane had led to the dissolution of some NQ (most probably, NQ incorporated into the BW outer sheath). Additional evidence that the outer sheath was composed of BW provided by the observation via TEM of the core–sheath fibers after extraction with hexane.

As seen from Figure 2, the extraction with THF led to an additional decrease in the mean fiber diameter and to the observation via TEM of fibers without a core–sheath structure. An indication that the inner sheath of the fibers was composed of hydrophobic PLA was the fact that after the extraction with THF, the fibrous material became hydrophilic. This is evidence that it consisted of PEO and NQ. The theoretical weight loss calculated after extraction with THF, assuming that entire amount of PLA had been removed from the fibers, was 22%. The experimentally determined weight loss was 24%. This loss was most probably due to the dissolution of that part of NQ which was incorporated in the PLA sheath. Similar results were also obtained after the consecutive selective extraction with hexane and THF for PEO/PCL/BW/NQ and PEO/PLA/BW/CQ fibrous materials.

**Figure 3:**
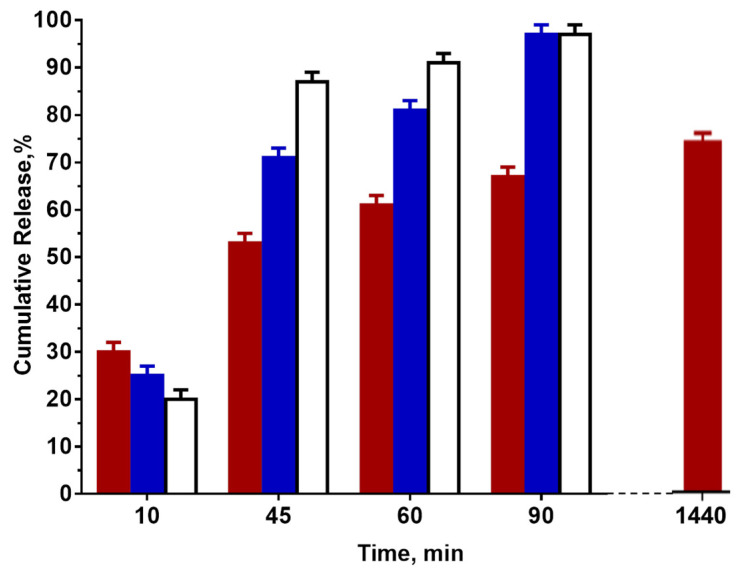
Release of NQ depending on the composition of the mat: PEO60/BW40/NQ (empty bar), PEO60/PCL20/BW20/NQ (blue bar), PEO60/PLA20/BW20/NQ (red bar).

The values of the released NQ (%) using a mat not containing polyester (core–sheath PEO60/BW40/NQ fibers), a PCL-containing mat (core–double sheath PEO60/PCL20/BW20/NQ fibers) and a PLA-containing mat (core–double sheath PEO60/PLA20/BW20/NQ) were compared (Figure 3). As observed, in the first stage of the release (5 min), the composition of the mat did not significantly alter the amount of released NQ and was lower than 30%. However, in the next stage (to 60 min), the composition of the mat considerably affected the amount of released NQ and the latter decreased in the following order: PEO60/BW40/NQ > PEO60/PCL20/BW20/NQ > PEO60/PLA20/BW20/NQ. This indicates that the presence of an inner sheath from the polyester in core–double sheath PEO60/PCL20/BW20/NQ and PEO60/PLA20/BW20/NQ fibers led to the slowing down of the NQ release in this time range, and the retardation was higher for the PEO60/PLA20/BW20/NQ mat as compared to the PEO60/PCL20/BW20/NQ mat. As seen in Figure 3, NQ was completely released from the PEO60/BW40/NQ and PEO60/PCL20/BW20/NQ mats after 90 min, while for PEO60/PLA20/BW20/NQ, the amount of released NQ was ca. 65%.

NQ was not completely released from the PEO60/PLA20/BW20/NQ mat even after 24 h, and the determined value of NQ released was ca. 75%. The obtained results revealed that the presence of a second inner sheath of polyester in core–double sheath fibers led to the slowing down of NQ release in the aqueous medium, and it depended on the polyester nature and molar mass. Considering that the sheath(s) in core–sheath and core–double sheath fibers were hydrophobic, an explanation for the registered NQ release data could be found in the differences in the behavior of the corresponding fibers in contact with water. The contact of the core–sheath PEO60/BW40/NQ fibers led to the fragmentation of the hydrophobic BW sheath due to PEO core swelling and dissolution, and to the release of the fibers’ internal content [15]. Therefore, the fastest NQ release in the case of core–sheath PEO60/BW40/NQ fibers could be attributed to the disintegration of the fibers in the aqueous medium. Therefore, PEO60/BW40/NQ mats might be used when a fast release of NQ is aimed at. We have already shown that the core–double sheath fibers from PEO60/PLA20/BW20/NQ preserve their structure and mean diameter value even after 48 h in an aqueous medium [16]. This indicates that these core–double sheath fibers have the advantage of being more stable in terms of integrity after contact with an aqueous medium as compared to the core–sheath ones. The results from the NQ release (Figure 3) indicate that the PLA and PCL inner sheath served as a barrier for the release of the drug, most probably due to the slowed down penetration of water molecules through the hydrophobic PLA or PCL inner and BW outer sheaths as compared to core–sheath PEO/BW/NQ fibers. It might be assumed that the more sustained release of NQ in the case of PLA could be related to the higher molar mass of PLA (ca. 259,000 g/mol) as compared to that of PCL (ca. 69,000 g/mol). The obtained results revealed that the drug release can be tuned via the selection of core–sheath or core–double sheath architecture of the fibers, as well as via selection of polyester for the formation of the inner sheath of the core–double sheath fibers PEO/polyester/BW/NQ.

### 3.2. Assessment of the Behavior of the Electrospun Materials in Contact with Phytopathogenic and Beneficial Microorganisms

#### 3.2.1. Assessment of the Behavior of the Electrospun Materials in Contact with Phytopathogenic Bacteria and Fungi

To the best of our knowledge, no data have been reported on the anti-microbial activity of NQ in contact with phytopathogenic microorganisms used in the present study except against *F. graminearum* [10]. For CQ, no information has been found on its activity in contact with the studied phytopathogens. The behavior of the individual compounds, NQ or CQ, in contact with the phytopathogens was assessed using the disk-diffusion method with filter paper disks preliminarily loaded with NQ or CQ. As seen from Figure S1a–c, the contact of NQ-loaded filter paper disks with the pathogenic microorganisms led to the formation of well-differentiated inhibition zones with diameters of 5.90 ± 0.20, 5.60 ± 0.40 and 5.20 ± 0.20 cm for *P. corrugata*, *F. graminearum* and *F. avenaceum*, respectively. This indicates that the individual compound NQ had very good anti-bacterial and anti-fungal activity against the studied phytopathogens. CQ-loaded filter paper disks exhibited a lower anti-bacterial activity against *P. corrugata* as seen from Figure S1d, and the registered inhibition zone was 1.90 ± 0.30 cm. This was attributed to the lower solubility of CQ as compared to that of NQ in an aqueous medium.

**Figure 4:**
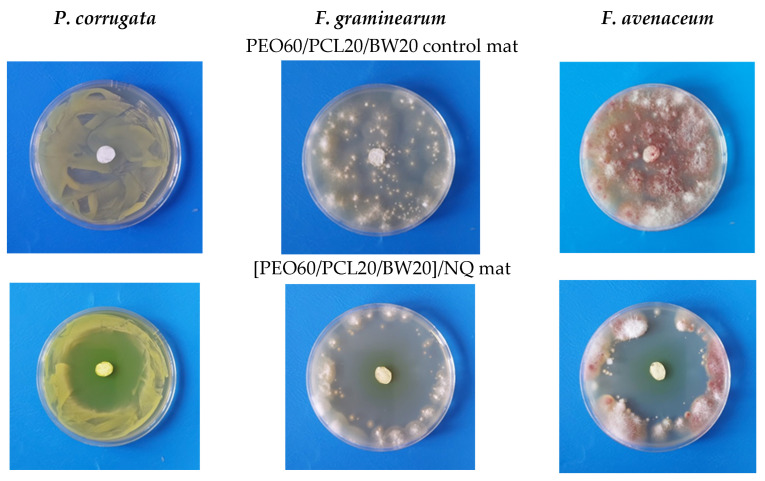
Digital images of the inhibition zones after contact of PEO60/PCL20/BW20/NQ mats with phytopathogenic microorganisms (lower row). Digital images of blank control PEO60/PCL20/BW20 mat after contact with the phytopathogenic microorganisms are presented for comparison (upper row). Left column: *P. corrugata*; middle column: *F. graminearum*; right column: *F. avenaceum*.

In the next step, the mats from the PEO/BW/NQ and PEO/PLA/BW/NQ series, as well as PEO/PCL/BW/NQ and PEO/PLA/BW/CQ mats, were subjected to microbiological tests for the assessment of their behavior in contact with the phytopathogenic bacterium *P. corrugata*, and the phytopathogenic fungi *F. graminearum* and *F. avenaceum*. Representative digital images of the inhibition zones in the case of the PEO60/PCL20/BW20/NQ mat are shown in Figure 4. As seen from Figure 4 in the upper row, and as registered for all non-NQ-containing control electrospun materials, no inhibition zone was observed for the PEO60/PCL20/BW20 blank control mat. This indicates that PEO, PCL and BW did not exhibit anti-bacterial and anti-fungal activity, and this result was expected since these components had no inherent bioactivity. The same results were obtained for the PEO/PLA/BW control mats. The presence of NQ in the PEO60/PCL20/BW20/NQ mats induced the appearance of well-differentiated inhibition zones (Figure 4, lower row). The inhibition zones values for all studied electrospun fibrous materials are listed in Table 1. As can be seen, for NQ-containing mats, the composition of the carrier did not significantly alter the diameter of the inhibition zone.

**Figure 5:**
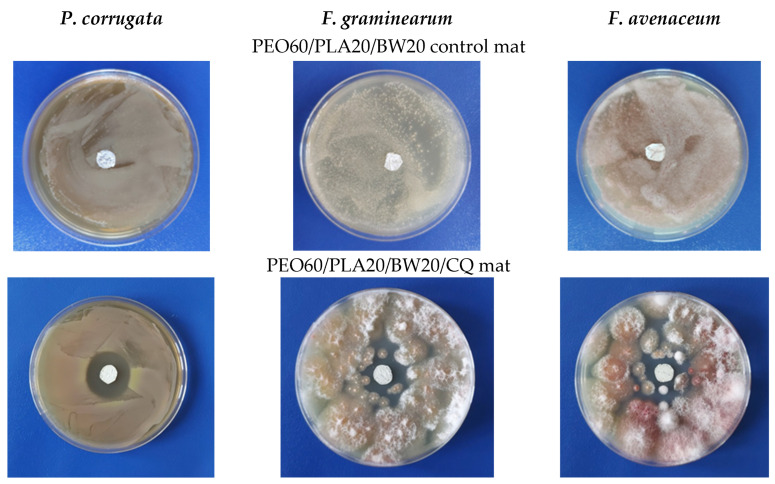
Digital images of the inhibition zones after contact of PEO60/PLA20/BW20/CQ mats with phytopathogenic microorganisms (lower row). Digital images of blank control PEO60/PLA20/BW20 after contact with the phytopathogenic microorganisms are presented for comparison (upper row). Left column: *P. corrugata*; middle column: *F. graminearum*; right column: *F. avenaceum*.

The smallest inhibition zones were registered for the PEO60/PLA20/BW20/CQ mats (Table 1, Figure 5). For these mats, the largest inhibition zone with a diameter of 2.70 ± 0.10 cm was detected after their contact with *P. corrugata*. This might be attributed to the poor solubility of CQ in an aqueous medium, which hampered CQ diffusion in the agar, and, respectively, the contact of CQ with the cells of the pathogenic microorganism.

#### 3.2.2. Assessment of the Behavior of the Electrospun Materials in Contact with Beneficial Bacteria and Fungi

There are no data on the behavior of NQ and CQ in contact with the beneficial microorganisms used in the present study —the bacteria *P. chlororaphis* and *B. amyloliquefaciens*, and the fungus *T. asperellum*. Therefore, at a first stage, the effect of NQ and CQ in contact with these microorganisms was tested using the disk-diffusion method with filter paper disks preliminarily loaded with NQ or CQ. NQ had anti-bacterial and antifungal activity against the studied beneficial microorganisms—well-differentiated inhibition zones with diameters of 4.40 ± 0.10, 4.60 ± 0.10 and 5.70 ± 0.10 cm for *P. chlororaphis*, *B. amyloliquefaciens* and *T. asperellum* were observed (Figure S2a–c). The anti-bacterial activity of CQ was weak and the inhibition zone measured 1.30 ± 0.10 and 1.80 ± 0.10 cm for *P. chlororaphis* and *B. amyloliquefaciens*, respectively (Figure S2d,e).

**Figure 6:**
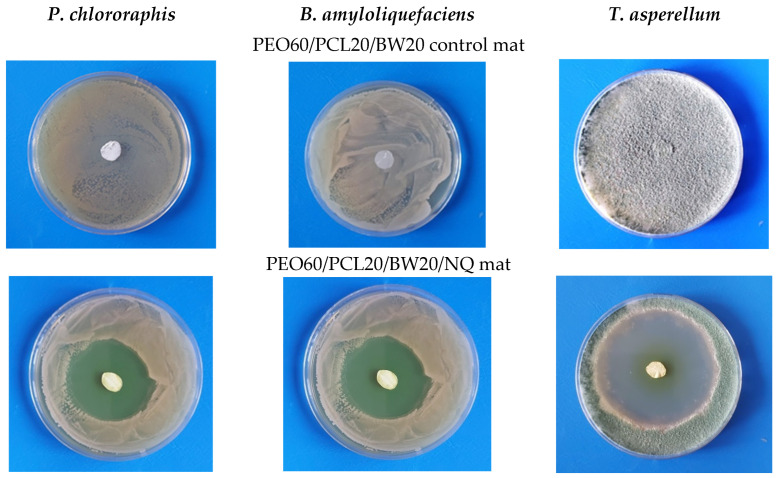
Digital images of the inhibition zones after contact of PEO60/PCL20/BW20/NQ mats with the beneficial microorganisms (lower row). Digital images of blank control PEO60/PCL20/BW20 mats after contact with the beneficial microorganisms (upper row). NQ content: 280 µg/disk. *P. chlororaphis* (left column), *B. amyloliquefaciens* (middle column), and *T. asperellum* (right column).

At a second stage, the mats from the PEO/BW/NQ and PEO/PLA/BW/NQ series, as well as the PEO/PCL/BW/NQ and PEO/PLA/BW/CQ mats, were subjected to microbiological tests for the assessment of their behavior in contact with the beneficial bacteria *P. chlororaphis* and *B. amyloliquefaciens* and the fungus *T. asperellum*. Representative digital images of the inhibition zones in the case of PEO60/PCL20/BW20/NQ mat are shown in Figure 6, lower row. As seen from Figure 6 in the upper row, and as registered for all of the studied blank control mats, they did not impede the development of the beneficial microorganisms.

The presence of NQ or CQ in the fibrous materials led to the appearance of inhibition zones. The inhibition zones’ values for all tested electrospun materials are listed in Table 2. As can be seen, the detected inhibition zones in the case of *T. asperellum* were larger than those for the *P. chlororaphis* and *B. amyloliquefaciens* bacteria. Concerning the composition of the fibrous carrier of NQ, there was no significant effect of the carrier composition on the diameter of the inhibition zones.

**Figure 7:**
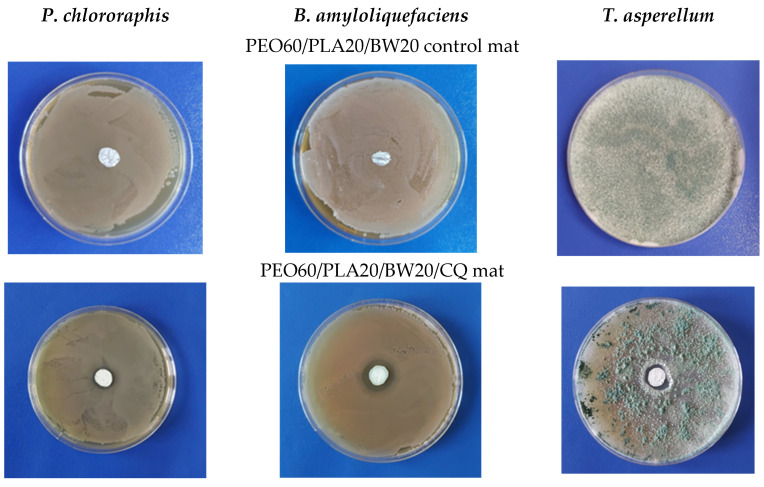
Digital images of the inhibition zones after contact of PEO60/PLA20/BW20/CQ mats with the beneficial microorganisms (lower row). Digital images of blank control PEO60/PLA20/BW20 after contact with the beneficial microorganisms are presented for comparison (upper row). NQ content: 280 µg/disk. *P. chlororaphis* (left column), *B. amyloliquefaciens* (middle column), and *T. asperellum* (right column).

The lowest diameters of the inhibition zones registered for the PEO60/PLA20/BW20CQ mats (Table 2, Figure 7) were attributed to the poor solubility of CQ in an aqueous medium, which slowed down the diffusion of CQ in the agar medium and the contact with the cells of the beneficial microorganisms.

The obtained results on the behavior of PEO/BW/NQ, PEO/PLA/BW/NQ, PEO/PCL/BW/NQ and PEO/PLA/BW/CQ mats in contact with the studied phytopathogenic or beneficial microorganisms revealed that the fibrous materials had anti-bacterial and anti-fungal activities. The main factor that affected the activity was the nature of the 8-hydroxyquinoline derivative used. The inhibition zones for NQ-containing fibrous materials were larger than those registered for CQ-containing mats. This was attributed to the poorer water solubility of CQ compared to that of NQ. It should be underlined that in the present study, the effect of NQ- or CQ-loaded mats on the beneficial microorganisms was evaluated. Noteworthy, the majority of the reports on new materials for plant protection evaluate the effect of the materials only on phytopathogens without considering their effect on beneficial microorganisms. The latter are of great importance for plant growth, and crop yield and quality. NQ and CQ had no selective activity—they do inhibit the growth both of phytopathogenic and beneficial microorganisms. The knowledge gained is of importance not only for the scientific community but also for those involved in the processes of plant production and protection. Therefore, new strategies and materials have to be developed in order to reach a balance between the inhibition of phytopathogens’ growth and the preservation of the viability of the beneficial microorganisms.

## 4. Conclusions

For the first time, the possibility of using electrospun materials consisting of core–sheath PEO/BW fibers or core–double sheath fibers from PEO/PLA/BW or PEO/PCL/BW loaded with the anti-bacterial and anti-fungal drugs NQ or CQ as agents for the control of phytopathogens, such as the bacterium *P. corrugata* and the fungi *F. graminearum* and *F. avenaceum*, which are widespread and cause significant damage to the yield of crop plants, was demonstrated. These fibrous materials might be applied via electrospinning on the green parts of plants, on plant wounds, and for seed coating. The microbiological tests on the behavior of NQ- or CQ-containing fibrous materials in contact with beneficial microorganisms (*P. chlororaphis*, *B. amyloliquefaciens* and *T. asperellum*) revealed that these mats exhibited anti-bacterial and anti-fungal activities as well. The results obtained are of importance regarding the applicability of this type of material and should be taken into account when the strategy of application of these fibrous materials in the agricultural field is considered.

## Figures and Tables

**Table 1:** Inhibition zones after contact of the electrospun materials with the corresponding phytopathogenic bacteria and fungi (NQ or CQ content in the fibrous materials—280 µg).

Fibrous Material	Inhibition Zone, cm		
	*P. corrugata*	*F. graminearum*	*F. avenaceum*
PEO/BW/NQ mat series			
PEO70/BW30/NQ	4.60 ± 0.14	4.40 ± 0.14	3.90 ± 0.13
PEO60/BW40/NQ	5.90 ± 0.12	4.50 ± 0.07	4.50 ± 0.13
PEO/PLA/BW/NQ mat series			
PEO70/PLA15/BW15/NQ	5.50 ± 0.14	4.80 ± 0.40	4.70 ± 0.12
PEO60/PLA20/BW20/NQ	5.40 ± 0.12	4.90 ± 0.18	5.10 ± 0.17
PEO60/PCL20/BW(20)/NQ mats			
PEO60/PCL20/BW20/NQ	5.30 ± 2.70	6.50 ± 5.80	6.00 ± 6.30
PEO60/PLA20/BW20/CQ mats			
PEO(60)/PLA(20)/BW(20)/CQ	2.70 ± 0.10	1.70 ± 0.30	1.60 ± 0.30

**Table 2:** Inhibition zones after contact of the electrospun materials with the corresponding beneficial microorganism (NQ or CQ content in the fibrous materials—280 µg).

Fibrous Material	Inhibition Zone, cm		
	*P. chlororaphis*	*B. amyloliquefaciens*	*T. asperellum*
PEO/BW/NQ mat series			
PEO70/BW30/NQ	4.00 ± 0.16	3.50 ± 0.04	5.00 ± 0.10
PEO60/BW40/NQ	4.20 ± 0.10	3.90 ± 0.10	5.70 ± 0.20
PEO/PLA/BW/NQ mat series			
PEO70/PLA15/BW15/NQ	3.90 ± 0.10	3.90 ± 0.10	5.60 ± 0.15
PEO60/PLA20/BW20/NQ	4.30 ± 0.10	4.00 ± 0.10	6.00 ± 0.10
PEO60/PCL20/BW(20)/NQ mats			
PEO60/PCL20/BW20/NQ	4.00 ± 1.10	4.00 ± 1.70	6.40 ± 0.10
PEO60/PLA20/BW20/CQ mats			
PEO(60)/PLA(20)/BW(20)/CQ	1.40 ± 0.10	1.70 ± 0.10	1.30 ± 0.10

## Data Availability

Not applicable.

## Supplementary Materials

The following supporting information can be downloaded at: https://www.mdpi.com/article/10.3390/ma16134882/s1, Figure S1: Digital images of the inhibition zones registered after contact of filter paper disks loaded with NQ (a–c) or CQ (d) with *P. corrugata* (a,d), *F. graminearum* (b), and *F. avenaceum* (c). NQ or CQ content: 280 µg/disk; Figure S2: Digital images of the inhibition zones registered after contact of filter paper disks loaded with NQ (a–c) or CQ (d,e) with *P. chlororaphis*, *B. amyloliquefaciens*, and *T. asperellum*. NQ or CQ content: 280 µg/disk.
