# Supplementary material for: Electrospun Fibers of Biocompatible and Biodegradable Polyesters, Poly(Ethylene Oxide) and Beeswax with Anti-Bacterial and Anti-Fungal Activities
Source: Materials (Basel). 2023 Jul 7;16(13):4882. doi: 10.3390/ma16134882 (PMC10343633; doi:10.3390/ma16134882)
Supplement: Supplementary file 1 [file materials-16-04882-s001.zip › materials-2480589-supplementary.pdf]

Supplementary Materials

Electrospun Fibers of Biocompatible and Biodegradable Polyesters, Poly(Ethylene Oxide) and Beeswax with Anti-Bacterial and Anti-Fungal Activities

Selin Kyuchyuk, Dilyana Paneva,* Nevena Manolova, Iliya Rashkov,* Daniela Karashanova, Mladen Naydenov, Nadya Markova

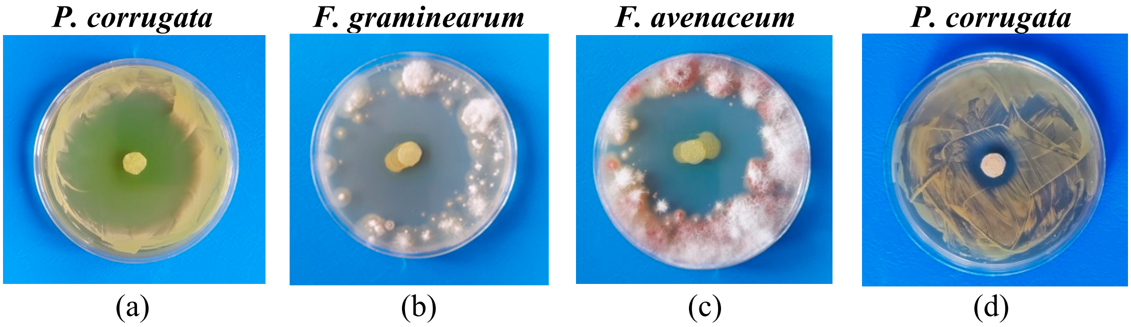

Figure S1. Digital images of the inhibition zones registered after contact of filter paper disks loaded with NQ (a-c) or CQ (d) with *P. corrugata* (a, d), *F. graminearum* (b), and *F. avenaceum* (c). NQ or CQ content: 280 μg/disk.

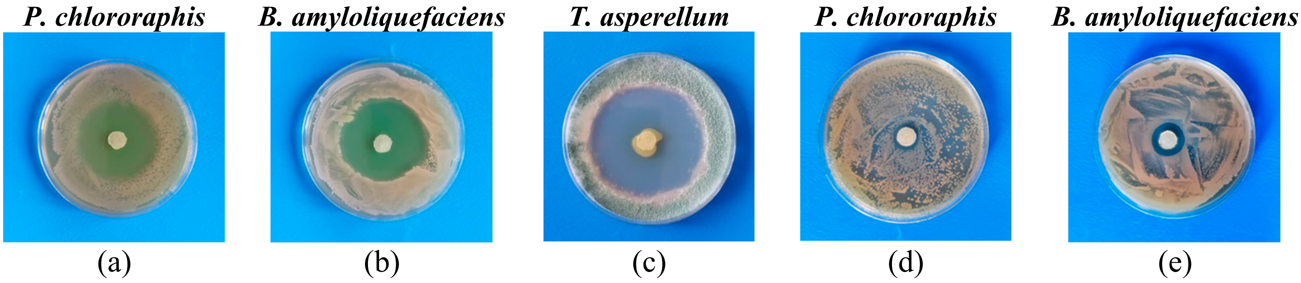

Figure S2. Digital images of the inhibition zones registered after contact of filter paper disks loaded with NQ (a-c) or CQ (d, e) with *P. chlororaphis*, *B. amyloliquefaciens*, and *T. asperellum*. NQ or CQ content: 280 μg/disk.
